# Effect of ERAS-based refined nursing on postoperative pain management in lung cancer surgery patients

**DOI:** 10.3389/fsurg.2026.1808117

**Published:** 2026-05-28

**Authors:** Jing Zhang, Bin Wang, Xiangnan Li, Fang Qi, Yan Wang

**Affiliations:** Department of Thoracic Surgery, Tangshan People’s Hospital (Tangshan Tumor Hospital), Tangshan, Hebei, China

**Keywords:** enhanced recovery after surgery, lung cancer surgery, opioid consumption, postoperative pain, refined nursing, rescue analgesia

## Abstract

**Background:**

Postoperative pain after lung cancer surgery can delay mobilization and recovery. We evaluated whether ERAS based refined nursing is associated with improved early pain recovery and reduced opioid use.

**Methods:**

In this retrospective cohort study at Tangshan People's Hospital, consecutive lung cancer surgery patients from October 2024 to October 2025 received ERAS based refined nursing or routine care. The primary outcome was pain trajectory from postoperative day zero to day three. Secondary endpoints included the area under the pain curve, morphine milligram equivalents, rescue analgesia, time to ambulation, length of stay, and short-term safety outcomes. The pain trajectory was assessed using a linear mixed-effects model. Multivariable linear regression models were used for continuous outcomes, and logistic regression models were fitted for binary outcomes.

**Results:**

Among 164 patients, 70 received ERAS-based refined nursing and 94 received routine care. The ERAS group showed lower pain scores from postoperative day 1 to day 3, lower opioid consumption, and fewer rescue analgesia events. In total-effect models, the group-by-time interaction for pain trajectory was significant, indicating faster pain recovery in the ERAS group. ERAS was also associated with lower cumulative pain burden, lower opioid consumption, shorter postoperative length of stay, and lower odds of rescue analgesia. Assessed safety outcomes were similar between groups.

**Conclusions:**

ERAS-based refined nursing was associated with faster early pain recovery, lower opioid requirements, and shorter hospitalization after lung cancer surgery.

## 1 Introduction

Lung cancer surgery is increasingly performed via minimally invasive techniques, but postoperative pain is still common and has meaningful clinical consequences. Typically, patients experience moderate to severe pain during the early post-operative period, even after VATS (Video-Assisted Thoracoscopic Surgery), and this can limit their ability to take deep breaths, the effectiveness of coughing, and moving around, which leads to poor pulmonary rehabilitation, as well as slower development of milestones. The recent thoracic surgery literature consistently reports that pain following minimally invasive pulmonary resection is not insignificant, and indeed may last beyond the early postoperative period for a nontrivial subset of patients, with acute pain intensity being repeatedly recognized as a major predictor for early persistent pain and long-term pain trajectories (1–3). These observations matter because pain control is not only a comfort outcome. It is tightly linked to functional recovery pathways that are central to lung resection care, including ambulation, chest physiotherapy participation, and readiness for discharge.

Concurrently, opioid exposure is also emerging as a metric for quality and safety in thoracic surgery. Opioids are still extensively utilized for perioperative analgesia, but excessive opioid consumption may be related to adverse events and delayed recovery, and contemporary thoracic series have described associations between in-hospital opioid use and postoperative complications (4). More generally, perioperative research is trending in the direction of opioid-sparing strategies and structured multimodal analgesia bundles, emphasizing a transition away from mere pain management toward an optimization of recovery via attenuation of opioid-related harms (5, 6). Specific to thoracic surgery, there is an increasing focus on modalities and pathways to decrease opioid use without compromising analgesia, with opioid-free anesthesia studies in thoracoscopic lung resection, and related perioperative programs aimed at reducing opioid usage while sustaining outcomes and safety (7, 8). These advances highlight the real practical requirement for ward-based approaches that can adapt multimodal principles to everyday bedside care.

Enhanced Recovery After Surgery programs were developed to address precisely this challenge by integrating evidence based perioperative practices into standardized pathways. The thoracic ERAS protocols promote integrated perioperative care, early mobilization, and multimodal analgesia to enhance recovery efficiency and to reduce complications and LOS (Length of Stay). A secondary finding of implementation research in thoracic surgery was that ERAS success relied not on protocol elements alone, but also on adherence, team coordination, and mode of delivery of pathway components in a manner that was both uniform and assessable (9, 10). In practice, nursing teams often become the key executors of pathway fidelity because nurses coordinate symptom monitoring, patient education, mobilization coaching, respiratory training support, and timely escalation of analgesic plans. Nurse led multidisciplinary cooperation models and refined nursing approaches have therefore emerged as promising implementation strategies to convert ERAS principles into daily ward routines (11–13). Several recent reports focused on refined nursing within an ERAS framework have described improvements in postoperative recovery indicators and patient reported outcomes in lung cancer surgical populations, suggesting that nursing process optimization can be an effective and feasible lever for ERAS delivery (12, 13).

Despite the growing adoption of thoracic ERAS, important evidence gaps remain for pain focused outcomes. Many ERAS evaluations prioritize LOS, complication rates, and cost, while pain is often treated as a secondary endpoint or summarized by single time point comparisons that can miss clinically important differences in recovery rate. In thoracic surgery, pain is inherently time dependent, with a steep early period followed by gradual decline, and interventions may primarily change the slope of recovery rather than the peak. This makes analytic approaches that account for repeated measures and within patient correlation particularly relevant. Moreover, real-world analgesia reflects both care processes and co-interventions, including regional techniques and NSAIDs (Nonsteroidal Anti-Inflammatory Drugs). Contemporary thoracic analgesia research highlights that regional techniques and multimodal strategies can reduce opioid use and influence pain outcomes, but the magnitude and generalizability vary across settings and surgical platforms such as thoracoscopy and robotic approaches (14–16). These considerations suggest that observational evaluations of ERAS based nursing should use modeling strategies that can separate time effects from group effects, incorporate clinically relevant covariates, and present effect estimates with uncertainty rather than relying solely on simple group comparisons.

Another gap relates to the outcomes selected to represent pain management quality. Dichotomous endpoints such as whether a patient ever exceeded a pain threshold can be insensitive in the setting of high POD0 pain and ceiling effects, whereas measures of overall pain burden and escalation needs may better reflect the clinical workload and patient experience. Recent thoracic pain research has emphasized the relevance of early persistent pain phenotypes after VATS and the need to focus on outcomes that capture persistence and functional relevance rather than isolated measurements (1, 3). In parallel, opioid consumption and rescue analgesia represent pragmatic and clinically important outcomes because they reflect real treatment demands and potential safety consequences. Studies of anesthetic and analgesic strategies in thoracoscopic lung resection increasingly report opioid use as a core outcome, supporting its value as a pathway endpoint for ERAS related nursing interventions (7, 8, 14).

Within this context, there is a clear need for evidence that evaluates ERAS based refined nursing in thoracic surgery using outcome definitions and statistical methods that match the clinical reality of pain recovery. The present study addresses this need in several ways. First, it considers pain as a short-term recovery trajectory and assesses it on multiple PODs (Postoperative Days), rather than relying on a one-time view. This follows the relation between pain management after lung cancer surgery and the major clinical target of postoperative recovery. Second, it combines the pain outcomes with opioid use and rescue analgesia. In this way, it connects patient-reported experience with treatment intensity and the potential risk related to opioids. Third, it examines recovery outcomes that are mechanistically tied to pain control, including ambulation timing and LOS, while also tracking common postoperative adverse events to contextualize safety. Fourth, it applies a modeling strategy designed for repeated pain measures and real-world confounding, using a linear mixed effects model to quantify differential pain change over time between groups, and multivariable regression models for cumulative pain burden, opioid requirements, and LOS, alongside logistic models for clinically meaningful binary endpoints. This approach is responsive to current implementation and outcomes research priorities in thoracic ERAS, which emphasize compliance, measurable effect estimates, and generalizable pathway components (9, 10, 17).

This study is also timely from an implementation perspective. Thoracic ERAS programs are expanding across diverse hospital settings, and recent literature stresses that pathway effectiveness depends on practical strategies that improve adherence and reduce variation. Refined nursing interventions are attractive because they can be deployed at scale, rely on structured workflows, and directly address day to day determinants of pain recovery such as assessment regularity, patient engagement, mobilization coaching, and coordination of multimodal analgesia. Evidence summaries focused on pain management for thoracoscopic lung cancer surgery further emphasize the importance of integrating analgesic strategies with perioperative care processes, a domain where nursing implementation is essential (16). By evaluating ERAS based refined nursing in a real-world thoracic surgery cohort and aligning outcomes with clinically meaningful pain and opioid endpoints, the study contributes practical evidence to guide pathway refinement and support data informed quality improvement.

Accordingly, the aim of this study was to evaluate the effect of ERAS based refined nursing on postoperative pain management among lung cancer surgery patients, with a primary focus on pain recovery over POD0 to POD3. Secondary objectives were to compare overall pain burden, opioid consumption, rescue analgesia, and key recovery outcomes including early ambulation and LOS, while assessing short term safety outcomes. We hypothesized that ERAS based refined nursing would be associated with a faster pain recovery trajectory and reduced opioid requirements, reflecting the combined effect of standardized nursing processes and coordinated multimodal analgesia within a thoracic ERAS pathway (11–13).

## 2 Materials and methods

### 2.1 Study design, setting, and study period

**Figure 1:**
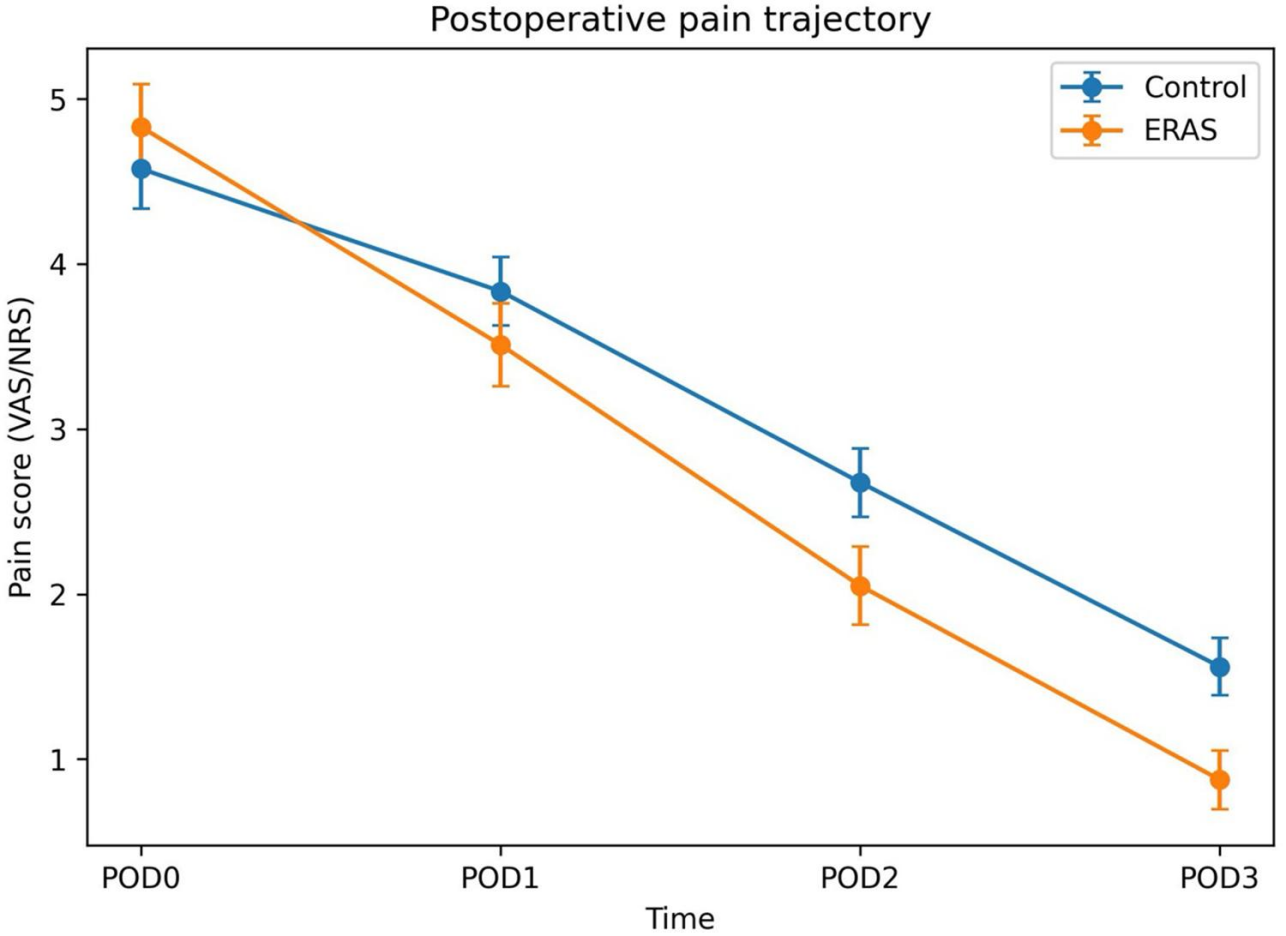
Flow diagram of patient selection and group allocation.

This retrospective cohort study was performed in the Department of Thoracic Surgery, Tangshan People's Hospital. Consecutive eligible patients who underwent lung cancer surgery between October 2024 and October 2025 were retrospectively reviewed. The ERAS-based refined nursing pathway was formally implemented in May 2025; patients treated before this time received routine perioperative nursing, whereas those treated thereafter received ERAS-based refined nursing. The flow of the study and patient selection are shown in Figure 1.

### 2.2 Participants

Patients who received elective pulmonary resection for lung cancer and had pain evaluations recorded from POD0 to POD3 were considered as candidates. However, patients were excluded if data regarding major exposures or primary outcomes were incomplete, if the procedure was not an eligible procedure for the surgical cohort, or if major covariates for adjustment could not be determined from clinical records. The final analysis cohort consisted of 164 patients, with 94 in the control and 70 in the ERAS group, as shown in Figure 1.

### 2.3 Exposure definition

The exposure of interest was receipt of ERAS-based refined nursing, defined as management under a structured perioperative nursing pathway that standardizes patient education, pain assessment and documentation, coordination of multimodal analgesia, early mobilization coaching, and respiratory rehabilitation support. Patients receiving routine perioperative nursing care outside the refined pathway were categorized as controls.

### 2.4 Data collection and covariates

The information was obtained from electronic medical records and nursing charts with the use of a case report form. Baseline characteristics were age, sex, BMI (body mass index), Charlson comorbidity index, smoking status, preoperative pain score, ASA (American Society of Anesthesiologists) class, and most relevant comorbidities. For the perioperative stage, the following variables were used and analyzed: surgical approach, type of resection, operation duration, approximated blood loss, regional analgesia, patient-controlled analgesia, and usage of NSAIDs.

### 2.5 Pain assessment and recording rule

Postoperative pain was assessed using the ward's routine 0–10 numeric rating scale documented by trained nurses as part of standard postoperative monitoring. Pain scores were recorded once daily from POD0 to POD3. For POD0, the pain score was documented after transfer to the ward and completion of immediate postoperative stabilization. For POD1–3, pain was recorded at a consistent daily time point during routine nursing assessment. When multiple pain entries were available within the same postoperative day, the value used for analysis followed the rule: the highest recorded pain score for that day was selected to reflect clinically relevant pain burden and to align with rescue analgesia decision-making in routine practice. If a rescue analgesic intervention occurred, pain assessments recorded after the intervention were not used to replace the daily value unless they represented the highest documented score within that day.

### 2.6 Outcomes

The primary endpoint was the postoperative pain trajectory from POD0 to POD3. Moderate-to-severe pain on POD2–POD3 was defined as a pain score of at least 4 on POD2 or POD3 and was assessed on POD2–POD3 only to reduce the influence of the immediate postoperative peak on POD0 and mitigate ceiling effects. Secondary outcomes were AUPC (the Area Under the Pain Curve) from POD0 to POD3 calculated with the trapezoidal rule, the peak pain score during the postoperative period, the time to pain score at most three, the incidence of moderate-to-severe pain defined as any pain score ≥4 at POD0, POD1, POD2, or POD3, the total opioid consumption expressed in MME (morphine milligram equivalents), the use of rescue analgesia, the time to ambulation, chest tube duration, postoperative LOS, and short-term safety outcomes including PONV (postoperative nausea and vomiting), pulmonary complications, ICU (intensive care unit) admission, and sleep disturbance.

### 2.7 ERAS-based refined nursing pathway components

The refined nursing path based on ERAS principles was applied in a standardized bundle, focusing on pain recovery, opioid reduction, early mobilization, and functional rehabilitation. It consisted of structured perioperative information on anticipated pain trajectory, coping strategies, and mobilization goals and included protocolized daily pain monitoring with predefined escalation criteria and timely communication with the surgical and anesthesia teams in case of insufficient analgesia. Nurses administered multimodal analgesia within institutional standards of care, providing nonopioid analgesics on a regular schedule unless contraindicated and supplemented with regional analgesia or patient-controlled analgesia protocols. As clinically appropriate, early mobilization instructions were delivered at the bedside, and progressive activity goals were set beginning on POD1. Respiratory rehabilitation support was provided by instruction on incentive spirometry and effective coughing to enhance ventilation. Furthermore, symptom co-management protocols were activated with proactive monitoring and nursing interventions for nausea, vomiting, sleep disturbance, and constipation symptoms that may enhance pain perception and hinder mobilization. The core components, responsible personnel, documentation sources, and retrospectively assessed delivery frequencies of the ERAS-based refined nursing pathway are summarized in Supplementary Table S8.

### 2.8 Statistical analysis

Continuous data are presented as the mean ± standard deviation (SD) when data were approximately normally distributed or as median [interquartile range (IQR)] otherwise. The categorical data are presented as the count and percentage. Between-group analysis for continuous variables was carried out using the approximate normal distribution Welch's *t* test; otherwise, the nonparametric Mann–Whitney U test was applied. The comparisons of categorical variables were performed by the *χ*^2^ test or Fisher's exact test when the expected cell counts were less than five. In this research, statistical tests were two-sided, and a *p*-value of less than 0.05 was regarded as statistically significant. Nevertheless, no prospective sample size calculation was performed because of the retrospective design, and the sample size reflected all eligible patients during the study period.

Pain trajectories from POD0 to POD3 were analyzed using a LMM (linear mixed-effects model) with a random intercept for patient to account for within-patient correlation across repeated measurements. Fixed effects included group, time, and the group by time interaction. Covariates were selected according to the total-effect and exploratory direct-effect modeling strategy described below. The primary inference for the pain trajectory analysis was based on the group by time interaction term. As a sensitivity analysis for the repeated-measures pain model, a random intercept and random slope specification was additionally fitted, and AIC/BIC values were compared with those of the primary random-intercept model.

AUPC was analyzed using multivariable OLS (Ordinary Least Squares) regression with heteroskedasticity robust standard errors. Total opioid consumption was analyzed using multivariable OLS regression with the outcome defined as log of MME plus one to reduce right skew. Postoperative LOS was analyzed using multivariable OLS regression with log transformed LOS. For binary outcomes, rescue analgesia and moderate-to-severe pain on POD2–POD3, defined as a pain score of at least 4 on POD2 or POD3, multivariable logistic regression was used, and effect estimates were reported as odds ratios with 95% confidence intervals.

To address the possibility that postoperative NSAIDs use and regional analgesia may function as mediators rather than baseline confounders, we fitted two levels of multivariable adjustment for outcome models. Total-effect models were adjusted for baseline and perioperative confounders only, including age, sex, BMI, preoperative pain score, operation time, ASA class, smoking status, surgical approach, and resection type. Exploratory direct-effect models additionally included NSAIDs use and regional analgesia.

As an additional sensitivity analysis, propensity score-based inverse probability of treatment weighting (IPTW) was performed to further assess the robustness of the main findings. Propensity scores were estimated using a logistic regression model including baseline and preoperative variables: age, sex, BMI, smoking status, ASA class, hypertension, diabetes, COPD, Charlson comorbidity index, preoperative pain score, preoperative analgesic use, surgical approach, and resection type. Stabilized IPTW weights were applied. Covariate balance before and after weighting was assessed using standardized mean differences. Weighted generalized estimating equations were used for the repeated pain trajectory analysis, and weighted regression models were used for secondary outcomes.

## 3 Results

### 3.1 Patient characteristics

There were 94 patients in the control group and 70 in the enhanced recovery after surgery (ERAS) based refined nursing group for lung cancer surgery. Baseline demographics were generally similar for the two groups; age, BMI, Charlson comorbidity index, and preoperative pain score were not significantly different between groups. The flow of the study and patient selection are shown in Figure 1. The distributions of sex, ASA class, and comorbidities were comparable. Smoking status seemed to verge on being unevenly distributed between the groups (Table 1).

**Table 1:** Baseline characteristics of lung cancer surgery patients in the ERAS and control groups.

Variable	Control (*n* = 94)	ERAS (*n* = 70)	*p* value
Age, years	62 ± 8	64 ± 9	0.403^a^
BMI, kg/m²	23.9 ± 2.8	24.0 ± 3.3	0.909^a^
Charlson comorbidity index	1.0 ± 0.8	1.1 ± 1.0	0.643^b^
Preoperative pain score	1.2 ± 1.0	1.3 ± 1.1	0.653^b^
Sex			0.403^c^
Female	37 (39.4%)	33 (47.1%)	
Male	57 (60.6%)	37 (52.9%)	
Smoking status			0.087^c^
Never	34 (36.2%)	28 (40.0%)	
Former	30 (31.9%)	30 (42.9%)	
Current	30 (31.9%)	12 (17.1%)	
ASA class			0.665^c^
I	31 (33.0%)	27 (38.6%)	
II	55 (58.5%)	36 (51.4%)	
III	8 (8.5%)	7 (10.0%)	
Hypertension			0.237^c^
No	42 (44.7%)	24 (34.3%)	
Yes	52 (55.3%)	46 (65.7%)	
Diabetes			0.827^c^
No	81 (86.2%)	62 (88.6%)	
Yes	13 (13.8%)	8 (11.4%)	
COPD			0.121^c^
No	81 (86.2%)	66 (94.3%)	
Yes	13 (13.8%)	4 (5.7%)	
Preoperative analgesic use			0.403^c^
No	89 (94.7%)	63 (90.0%)	
Yes	5 (5.3%)	7 (10.0%)	

a Continuous variable are presented as mean ± SD unless otherwise specified; categorical variables are presented as *n* (%).

b Between-group comparisons for continuous variables used Welch's *t*-test for approximately normal distributions and Mann–Whitney U test for non-normal distributions.

c Between-group comparisons for categorical variables used *χ*² test; Fisher's exact test was used when expected cell counts were <5.

### 3.2 Surgical characteristics and perioperative analgesia-related variables

Surgical characteristics were generally comparable between groups. Operation time and estimated blood loss did not differ significantly. The proportions of VATS vs. open approach and resection type were also similar. Use of regional analgesia and PCA (Patient Controlled Analgesia) did not significantly differ. NSAIDs use showed a borderline trend toward higher utilization in the ERAS group (Table 2).

**Table 2:** Surgical characteristics and perioperative analgesia-related variables in the ERAS and control groups.

Variable	Control (*n* = 94)	ERAS (*n* = 70)	*p* value
Operation time, min	150 ± 27	154 ± 31	0.427^a^
Estimated blood loss, mL	82 (56, 126)	80 (55, 104)	0.581^a^
Surgical approach			0.198^b^
Open	14 (14.9%)	5 (7.1%)	
VATS	80 (85.1%)	65 (92.9%)	
Resection type			0.609^b^
Lobectomy	78 (83.0%)	55 (78.6%)	
Segmentectomy	16 (17.0%)	15 (21.4%)	
Regional analgesia			0.245^b^
No	46 (48.9%)	27 (38.6%)	
Yes	48 (51.1%)	43 (61.4%)	
Patient-controlled analgesia (PCA)			0.469^b^
No	40 (42.6%)	25 (35.7%)	
Yes	54 (57.4%)	45 (64.3%)	
NSAIDs use			0.053^b^
No	36 (38.3%)	16 (22.9%)	
Yes	58 (61.7%)	54 (77.1%)	

a Between-group comparisons for continuous variables used Welch's *t*-test for approximately normal distributions and Mann–Whitney U test for non-normal distributions.

b Between-group comparisons for categorical variables used *χ*² test; Fisher's exact test was used when expected cell counts were <5.

### 3.3 Postoperative pain outcomes

**Figure 2:**
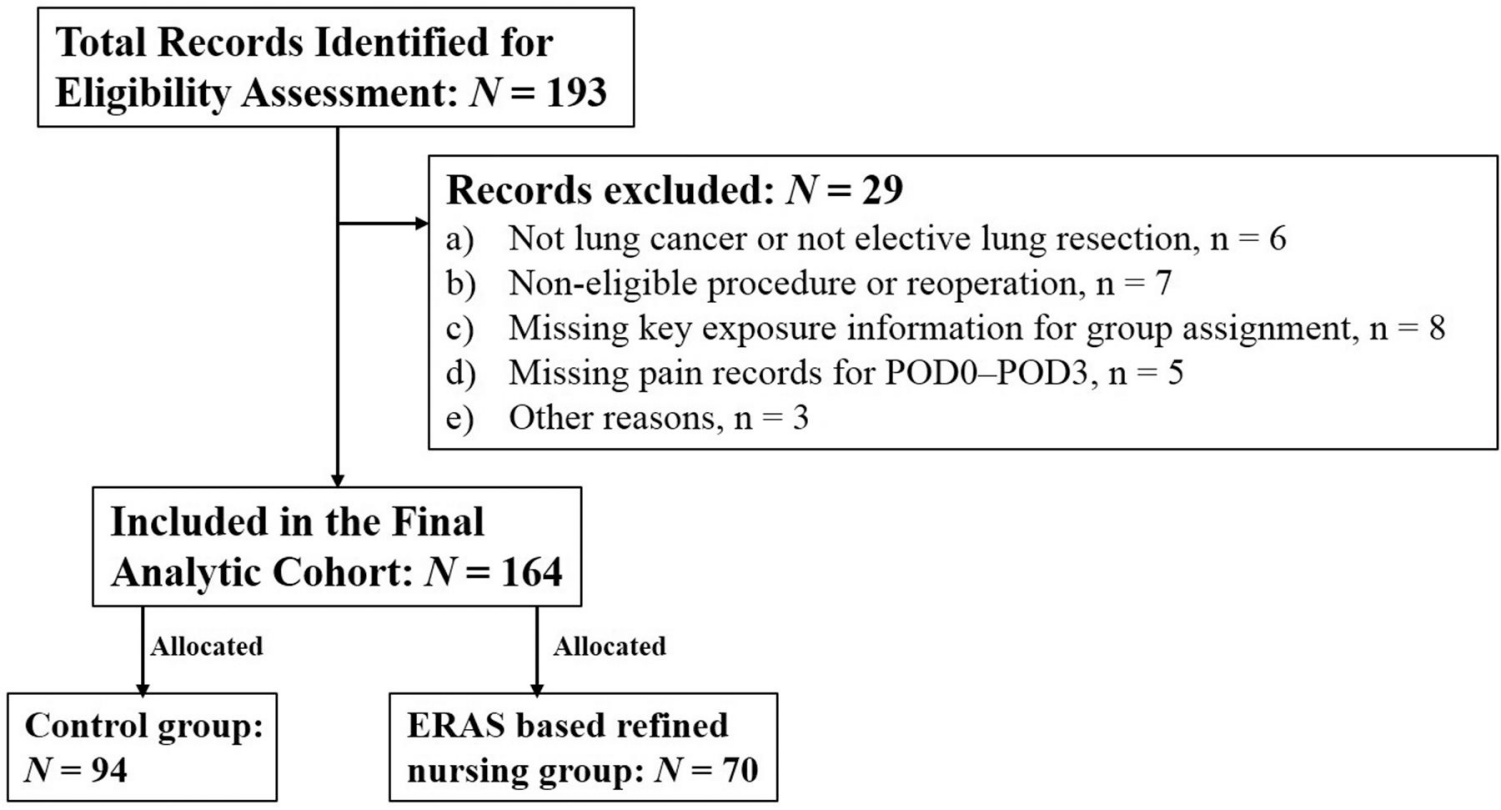
Postoperative pain trajectories from POD0 to POD3 in the ERAS and control groups.

Postoperative pain scores showed a time-dependent between-group separation. Pain on POD0 was comparable, whereas from POD1 onward the ERAS group demonstrated lower pain scores, with a larger separation on POD2 and POD3 (Table 3). Postoperative pain trajectories from POD0 to POD3 are shown in Figure 2. In unadjusted analyses, the ERAS group had a lower overall pain burden as reflected by a lower AUPC from POD0 to POD3. The time to achieve a pain score ≤3 was shorter in the ERAS group. In contrast, the proportion of patients experiencing moderate-to-severe pain defined as any pain score ≥4 across POD0–POD3 did not differ between groups.

**Table 3:** Postoperative pain outcomes from POD0 to POD3 in the ERAS and control groups.

Variable	Control (*n* = 94)	ERAS (*n* = 70)	*p* value
Pain score on POD0	4.6 ± 1.2	4.8 ± 1.1	0.162^c^
Pain score on POD1	3.8 ± 1.0	3.5 ± 1.1	0.047^c^
Pain score on POD2	2.7 ± 1.0	2.0 ± 1.0	<0.001^c^
Pain score on POD3	1.6 ± 0.9	0.9 ± 0.8	<0.001^c^
Peak postoperative pain score	4.7 ± 1.1	4.9 ± 1.0	0.312^c^
AUPC^a^ (POD0–POD3)	9.58 ± 2.58	8.41 ± 2.49	0.004^c^
Time to pain score ≤ 3, days	2.0 ± 1.0	1.7 ± 0.8	0.020^c^
Moderate-to-severe pain^b^ (any time, ≥4)			0.866^d^
No	21 (22.3%)	14 (20.0%)	
Yes	73 (77.7%)	56 (80.0%)	

a AUPC (area under the pain curve) from POD0–POD3 was calculated using the trapezoidal rule: AUPC = [(P0 + P1)/2] + [(P1 + P2)/2] + [(P2 + P3)/2].

b Moderate-to-severe pain was defined as any postoperative pain score ≥4.

c Between-group comparisons for continuous variables used Welch's *t*-test for approximately normal distributions and Mann–Whitney U test for non-normal distributions.

d Between-group comparisons for categorical variables used *χ*² test; Fisher's exact test was used when expected cell counts were <5.

### 3.4 Analgesic consumption and rescue analgesia

The total dose of postoperative opioids was significantly lower in the ERAS group. In addition, the number of patients needing rescue analgesia was lower in the ERAS group (Table 4). On the other hand, although the time to first rescue analgesia was numerically longer in the ERAS group among patients who received this intervention, the difference was not statistically significant.

**Table 4:** Analgesic consumption and rescue analgesia in the ERAS and control groups.

Variable	Control (*n* = 94)	ERAS (*n* = 70)	*p* value
Total opioid consumption, MME	24.9 ± 9.9	18.1 ± 9.7	<0.001^b^
Rescue analgesia^a^			0.036^c^
No	63 (67.0%)	58 (82.9%)	
Yes	31 (33.0%)	12 (17.1%)	
Time to first rescue analgesia, hours	10.9 (7.5, 14.5)	13.3 (9.5, 14.5)	0.386^b^

a Time to first rescue analgesia is presented as median (Q1, Q3) among patients who received rescue analgesia.

b Between-group comparisons for continuous variables used Welch's *t*-test for approximately normal distributions and Mann–Whitney U test for non-normal distributions.

c Between-group comparisons for categorical variables used *χ*² test; Fisher's exact test was used when expected cell counts were <5.

### 3.5 Recovery and safety outcomes

Patients in the ERAS group achieved ambulation earlier than those in the control group and had a shorter postoperative LOS (Table 5). Chest tube duration did not differ between groups. The incidences of postoperative nausea, pulmonary complications, ICU admission, and sleep disturbance were similar between groups.

**Table 5:** Recovery and safety outcomes in the ERAS and control groups.

Variable	Control (*n* = 94)	ERAS (*n* = 70)	*p* value
Time to ambulation, days	1.6 ± 0.6	1.0 ± 0.5	<0.001^b^
Chest tube duration, days	3.8 ± 1.1	3.8 ± 1.0	0.971^b^
Postoperative length of stay, days^a^	7.2 (6.5, 8.3)	6.3 (5.2, 7.4)	<0.001^b^
Sleep disturbance			0.520^c^
No	70 (74.5%)	56 (80.0%)	
Yes	24 (25.5%)	14 (20.0%)	
Postoperative nausea/vomiting (PONV)			0.244^c^
No	79 (84.0%)	64 (91.4%)	
Yes	15 (16.0%)	6 (8.6%)	
Pulmonary complications			0.303^c^
No	87 (92.6%)	68 (97.1%)	
Yes	7 (7.4%)	2 (2.9%)	
ICU admission			0.637^c^
No	91 (96.8%)	69 (98.6%)	
Yes	3 (3.2%)	1 (1.4%)	

a Postoperative length of stay is presented as median (Q1, Q3); other continuous variables are presented as mean ± SD. Categorical variables are presented as *n* (%).

b Between-group comparisons for continuous variables used Welch's *t*-test for approximately normal distributions and Mann–Whitney U test for non-normal distributions.

c Between-group comparisons for categorical variables used *χ*² test; Fisher's exact test was used when expected cell counts were <5.

### 3.6 Multivariable modeling

In the total-effect models adjusted for baseline confounders only, the Group × Time interaction for postoperative pain trajectory remained statistically significant, indicating a faster decline in pain scores over time in the ERAS group (*β* = −0.311, 95% CI −0.406 to −0.217, *p* < 0.001; Table 6; Supplementary Table S1). In these total-effect models, ERAS was also associated with a lower AUPC from POD0 to POD3 (*β* = −0.973, 95% CI −1.783 to −0.163, *p* = 0.019), lower log-transformed total opioid consumption (*β* = −0.415, 95% CI −0.668 to −0.162, *p* = 0.001), shorter log-transformed postoperative length of stay (*β* = −0.179, 95% CI −0.272 to −0.086, *p* < 0.001), and lower odds of rescue analgesia (OR = 0.35, 95% CI 0.15–0.81, *p* = 0.014) (Table 6; Supplementary Tables S2–S5). By contrast, the association between ERAS and moderate-to-severe pain on POD2–POD3 did not reach statistical significance in the total-effect model (OR = 0.29, 95% CI 0.05–1.64, *p* = 0.161; Table 6, Supplementary Table S6).

**Table 6:** Total-effect and exploratory direct-effect models of ERAS-based refined nursing across key postoperative outcomes.

Outcome	Model	Key effect	Effect size	95% CI	*p* value
Postoperative pain trajectory (POD0–POD3)	LMM (random intercept), total-effect model	Group × Time (interaction)	−0.311	−0.406–−0.217	<0.001
Postoperative pain trajectory (POD0–POD3)	LMM (random intercept), direct-effect model	Group × Time (interaction)	−0.311	−0.406–−0.217	<0.001
AUPC (POD0–POD3)	OLS (HC3 robust SE), total-effect model	Group	−0.973	−1.783–−0.163	0.019
AUPC (POD0–POD3)	OLS (HC3 robust SE), direct-effect model	Group	−0.607	−1.406–0.191	0.136
Total opioid consumption [log(MME+1)]	OLS (HC3 robust SE), total-effect model	Group	−0.415	−0.668–−0.162	0.001
Total opioid consumption [log(MME + 1)]	OLS (HC3 robust SE), direct-effect model	Group	−0.33	−0.565–−0.096	0.006
Postoperative length of stay [log(LOS)]	OLS (HC3 robust SE), total-effect model	Group	−0.179	−0.272–−0.086	<0.001
Postoperative length of stay [log(LOS)]	OLS (HC3 robust SE), direct-effect model	Group	−0.169	−0.259–−0.079	<0.001
Rescue analgesia (Yes vs. No)	Logistic regression, total-effect model	Group	0.35 (OR)	0.15–0.81	0.014
Rescue analgesia (Yes vs. No)	Logistic regression, direct-effect model	Group	0.37 (OR)	0.16–0.90	0.028
Moderate-to-severe pain on POD2–POD3 (≥4)	Logistic regression, total-effect model	Group	0.29 (OR)	0.05–1.64	0.161
Moderate-to-severe pain on POD2–POD3 (≥4)	Logistic regression, direct-effect model	Group	0.33 (OR)	0.05–2.41	0.276

Total-effect models were adjusted for age, sex, BMI, preoperative pain score, operation time, ASA class, smoking status, surgical approach, and resection type. Direct-effect models were additionally adjusted for postoperative NSAIDs use and regional analgesia, which may lie on the causal pathway between ERAS implementation and postoperative pain-related outcomes.

In exploratory direct-effect models additionally adjusted for NSAIDs use and regional analgesia, the Group × Time interaction for pain trajectory remained statistically significant (Table 6; Supplementary Table S1). The associations of ERAS with lower opioid consumption, shorter postoperative length of stay, and reduced odds of rescue analgesia also remained significant, whereas the association with AUPC was attenuated and no longer statistically significant after additional adjustment for these potential mediators (*β* = −0.607, 95% CI −1.406–0.191, p = 0.136) (Table 6; Supplementary Tables S2–S5). The association with moderate-to-severe pain on POD2–POD3 remained non-significant in the direct-effect model as well (OR = 0.33, 95% CI 0.05–2.41, *p* = 0.276; Table 6; Supplementary Table S6). A sensitivity analysis using a random intercept and random slope specification for pain trajectory yielded similar inferences. The alternative model showed a slightly lower AIC but a higher BIC than the primary random-intercept model (AIC 1,645.55 compared to 1,650.77, and BIC 1,739.76 compared to 1,736.01), supporting retention of the more parsimonious random-intercept specification (Supplementary Table S7).

In an additional IPTW sensitivity analysis, post-weighting covariate balance was acceptable, with all absolute standardized mean differences below 0.10 (Supplementary Table S9). The weighted analysis showed results consistent with the primary models. The ERAS group remained associated with a more favorable pain trajectory over time (Group × Time: *β* = −0.323, 95% CI −0.429 to −0.217, *p* < 0.001), lower AUPC (*β* = −0.955, 95% CI −1.727 to −0.183, *p* = 0.015), lower log-transformed opioid consumption (*β* = −0.419, 95% CI −0.666 to −0.172, *p* < 0.001), shorter log-transformed postoperative length of stay (*β* = −0.199, 95% CI −0.290 to −0.108, *p* < 0.001), and lower odds of rescue analgesia (OR = 0.31, 95% CI 0.14–0.70, *p* = 0.005). The association with moderate-to-severe pain on POD2–POD3 remained non-significant (OR = 0.28, 95% CI 0.05–1.52, *p* = 0.140) (Supplementary Table S10).

## 4 Discussion

Postoperative pain after lung cancer surgery remains a major barrier to early mobilization and effective pulmonary rehabilitation, and it can delay recovery even when minimally invasive techniques are used. In this thoracic surgery cohort, ERAS-based refined nursing showed a coherent pattern of benefit across pain recovery, opioid exposure, and LOS.

The most informative signal was the covariate-adjusted pain trajectory, where ERAS was independently associated with a more rapid decrease in pain scores from POD0 through to POD3. This trajectory-based result is stronger methodologically than single-time point comparisons and is consistent with core ERAS and ESTS (European Society of Thoracic Surgeons) principles that protocolized care, early mobilization, and multimodal analgesia lead to enhanced recovery (18).

The day-by-day pattern also supports a recovery rate interpretation. Pain scores were similar immediately after surgery but separated increasingly from POD1 onward, which is consistent with ERAS interventions that act through structured nursing processes, such as frequent assessment, timely titration of analgesia, education that improves coping and participation in breathing exercises, and early mobilization coaching. Thoracic ERAS studies and systematic evidence have reported reductions in LOS and improvements in short term outcomes after pathway implementation, supporting the plausibility of a time dependent recovery advantage rather than a simple shift in peak pain (19–21).

Opioid sparing is a clinically important outcome in thoracic surgery, where early recovery and respiratory function can be compromised by opioid related adverse effects. In this dataset, ERAS was independently associated with lower opioid requirements and lower odds of rescue analgesia after adjustment. This direction is consistent with thoracic ERAS implementation research that has shown meaningful reductions in postoperative opioid exposure when multimodal strategies are embedded into standardized pathways (21, 22). The broader thoracic ERAS literature also highlights the importance of reducing opioid exposure as part of recovery optimization, particularly as persistent opioid use after lung resection has become an increasing concern (23, 24).

The modeling results also underscore the contribution of multimodal analgesia components. Contemporary procedure specific recommendations for VATS emphasize baseline non-opioid analgesics when appropriate and regional analgesia techniques to reduce opioid requirements and improve recovery quality (25). Evidence syntheses comparing regional analgesia approaches in VATS similarly support the value of regional techniques in postoperative pain control (26). In our analysis, variables reflecting regional analgesia and NSAIDs use were associated with more favorable pain and opioid outcomes, which is consistent with the multimodal pathway concept and helps explain why bundled ERAS nursing may translate into measurable reductions in opioid needs.

In the present study, the association between ERAS and AUPC was statistically significant in the total-effect model but was attenuated after additional adjustment for NSAIDs use and regional analgesia in the exploratory direct-effect model (Table 6; Supplementary Table S2). This pattern suggests that part of the observed reduction in cumulative pain burden may be mediated through postoperative multimodal analgesic delivery rather than reflecting baseline confounding alone (18, 21).

Safety signals were also important for interpretation. There were no statistically significant between group differences in the assessed adverse outcomes in this cohort. Prior thoracic ERAS studies and systematic reviews generally support that protocolized recovery and opioid sparing can be achieved without clear increases in short term complications, although event rates and statistical power often limit precision (19–21).

This study has limitations. The retrospective single center design introduces the possibility of residual confounding and selection bias. ERAS implementation can coincide with temporal changes in surgical or perioperative practice, which may influence outcomes even after covariate adjustment. Pain outcomes were evaluated only in the early postoperative window, and longer follow up is needed to evaluate persistent postsurgical pain and longer-term opioid use, outcomes that are increasingly emphasized in thoracic recovery research (23, 25). Future prospective studies should include implementation fidelity measures, standardize pain assessment timing and context, and extend follow up to clarify which nursing driven components most strongly influence pain recovery and opioid exposure. In addition, several secondary binary endpoints, particularly moderate-to-severe pain on POD2–POD3, ICU admission, and pulmonary complications, may have been underpowered because of the limited number of events and should therefore be interpreted cautiously.

## 5 Conclusion

In this cohort of lung cancer surgery patients, ERAS-based refined nursing was associated with a faster early postoperative pain recovery trajectory from POD0 to POD3. In total-effect models, ERAS was also associated with lower cumulative pain burden, lower opioid consumption, fewer rescue analgesia events, and shorter postoperative hospitalization. No significant between-group differences were observed for the investigated short-term safety outcomes. These findings support the potential value of structured nursing implementation within thoracic ERAS pathways, while suggesting that part of the observed benefit may be mediated through postoperative multimodal analgesic strategies.

## Data Availability

The original contributions presented in the study are included in the article/Supplementary Material, further inquiries can be directed to the corresponding author.
