# Supplementary material for: Effect of ERAS-based refined nursing on postoperative pain management in lung cancer surgery patients
Source: Front Surg. 2026 May 28;13:1808117. doi: 10.3389/fsurg.2026.1808117 (PMC13254267; doi:10.3389/fsurg.2026.1808117)
Supplement: Supplementary file 1 [file Table1.docx]

**Supplementary Table S1.** Total-effect and exploratory direct-effect linear mixed-effects models for postoperative pain trajectory from POD0 to POD3.

| **Term** | **Estimate** | **SE** | **z** | **95% CI (low)** | **95% CI (high)** | **p value** |
| --- | --- | --- | --- | --- | --- | --- |
| Intercept | 5.25 | 0.3 | 17.27 | 4.65 | 5.84 | <0.001 |
| C(ASA)[T.2] | 0.27 | 0.2 | 1.39 | -0.11 | 0.66 | 0.166 |
| C(ASA)[T.3] | 0.59 | 0.31 | 1.9 | -0.02 | 1.21 | 0.057 |
| C(Smoking)[T.1] | 0.08 | 0.14 | 0.52 | -0.21 | 0.36 | 0.604 |
| C(Smoking)[T.2] | 0.18 | 0.16 | 1.13 | -0.13 | 0.49 | 0.259 |
| C(Surgical_Approach)[T.1] | -0.35 | 0.21 | -1.71 | -0.75 | 0.05 | 0.087 |
| C(Resection_Type)[T.2] | 0.01 | 0.16 | 0.03 | -0.31 | 0.32 | 0.974 |
| Group | 0.31 | 0.15 | 2.08 | 0.02 | 0.6 | 0.038 |
| Time0 | -1.02 | 0.03 | -32.2 | -1.08 | -0.96 | <0.001 |
| Group:Time0 | -0.31 | 0.05 | -6.42 | -0.41 | -0.22 | <0.001 |
| Age | -0.01 | 0.01 | -1.21 | -0.04 | 0.01 | 0.225 |
| BMI | -0.01 | 0.02 | -0.62 | -0.05 | 0.03 | 0.535 |
| Preop_Pain | 0.08 | 0.06 | 1.31 | -0.04 | 0.2 | 0.189 |
| Operation_Time | 0 | 0 | -1.26 | -0.01 | 0 | 0.208 |
| Regional_Analgesia | -0.57 | 0.12 | -4.6 | -0.81 | -0.33 | <0.001 |
| NSAIDs | -0.36 | 0.13 | -2.72 | -0.63 | -0.1 | 0.007 |
| Sex | -0.07 | 0.12 | -0.57 | -0.32 | 0.17 | 0.570 |
