# Supplementary material for: Effect of ERAS-based refined nursing on postoperative pain management in lung cancer surgery patients
Source: Front Surg. 2026 May 28;13:1808117. doi: 10.3389/fsurg.2026.1808117 (PMC13254267; doi:10.3389/fsurg.2026.1808117)
Supplement: Supplementary file 2 [file Table2.docx]

**Supplementary Table S2.** Total-effect and exploratory direct-effect multivariable OLS regression models for AUPC from POD0 to POD3.

| **Term** | **Estimate** | **SE (robust)** | **95% CI (low)** | **95% CI (high)** | **p value** |
| --- | --- | --- | --- | --- | --- |
| Intercept | 15.907 | 3.776 | 8.506 | 23.307 | <0.001 |
| C(ASA)[T.2] | 0.838 | 0.632 | -0.401 | 2.077 | 0.185 |
| C(ASA)[T.3] | 1.72 | 1.156 | -0.545 | 3.985 | 0.137 |
| C(Smoking)[T.1] | 0.296 | 0.488 | -0.66 | 1.252 | 0.544 |
| C(Smoking)[T.2] | 0.643 | 0.531 | -0.397 | 1.683 | 0.225 |
| C(Surgical_Approach)[T.1] | -0.97 | 0.578 | -2.104 | 0.163 | 0.093 |
| C(Resection_Type)[T.2] | 0.159 | 0.587 | -0.992 | 1.31 | 0.787 |
| Group | -0.607 | 0.407 | -1.406 | 0.191 | 0.136 |
| Age | -0.043 | 0.042 | -0.125 | 0.039 | 0.308 |
| Sex | -0.232 | 0.393 | -1.003 | 0.539 | 0.556 |
| BMI | -0.056 | 0.068 | -0.189 | 0.077 | 0.410 |
| Preop_Pain | 0.24 | 0.21 | -0.171 | 0.651 | 0.253 |
| Operation_Time | -0.007 | 0.008 | -0.022 | 0.009 | 0.417 |
| Regional_Analgesia | -1.741 | 0.425 | -2.574 | -0.907 | <0.001 |
| NSAIDs | -1.169 | 0.465 | -2.081 | -0.257 | 0.012 |
