# Supplementary material for: Effect of ERAS-based refined nursing on postoperative pain management in lung cancer surgery patients
Source: Front Surg. 2026 May 28;13:1808117. doi: 10.3389/fsurg.2026.1808117 (PMC13254267; doi:10.3389/fsurg.2026.1808117)
Supplement: Supplementary file 3 [file Table3.docx]

**Supplementary Table S3.** Total-effect and exploratory direct-effect multivariable OLS regression models for total opioid consumption.

| **Term** | **Estimate** | **SE (robust)** | **95% CI (low)** | **95% CI (high)** | **p value** |
| --- | --- | --- | --- | --- | --- |
| Intercept | 3.321 | 1.16 | 1.048 | 5.595 | 0.004 |
| C(ASA)[T.2] | 0.003 | 0.172 | -0.334 | 0.339 | 0.988 |
| C(ASA)[T.3] | -0.219 | 0.36 | -0.925 | 0.488 | 0.544 |
| C(Smoking)[T.1] | 0.061 | 0.142 | -0.218 | 0.34 | 0.668 |
| C(Smoking)[T.2] | 0.106 | 0.143 | -0.174 | 0.386 | 0.458 |
| C(Surgical_Approach)[T.1] | -0.272 | 0.215 | -0.694 | 0.15 | 0.206 |
| C(Resection_Type)[T.2] | 0.127 | 0.12 | -0.108 | 0.361 | 0.289 |
| Group | -0.33 | 0.12 | -0.565 | -0.096 | 0.006 |
| Age | 0.005 | 0.014 | -0.022 | 0.032 | 0.705 |
| Sex | 0.045 | 0.119 | -0.187 | 0.278 | 0.703 |
| BMI | 0.015 | 0.023 | -0.03 | 0.059 | 0.515 |
| Preop_Pain | 0.026 | 0.048 | -0.069 | 0.121 | 0.591 |
| Operation_Time | -0.002 | 0.002 | -0.007 | 0.002 | 0.334 |
| Regional_Analgesia | -0.427 | 0.104 | -0.631 | -0.223 | <0.001 |
| NSAIDs | -0.248 | 0.109 | -0.461 | -0.035 | 0.022 |
