# Supplementary material for: Effect of ERAS-based refined nursing on postoperative pain management in lung cancer surgery patients
Source: Front Surg. 2026 May 28;13:1808117. doi: 10.3389/fsurg.2026.1808117 (PMC13254267; doi:10.3389/fsurg.2026.1808117)
Supplement: Supplementary file 4 [file Table4.docx]

**Supplementary Table S4.** Total-effect and exploratory direct-effect multivariable OLS regression models for postoperative length of stay.

| **Term** | **Estimate** | **SE (robust)** | **95% CI (low)** | **95% CI (high)** | **p value** |
| --- | --- | --- | --- | --- | --- |
| Intercept | 2.203 | 0.314 | 1.589 | 2.818 | <0.001 |
| C(ASA)[T.2] | 0.07 | 0.075 | -0.077 | 0.216 | 0.351 |
| C(ASA)[T.3] | 0.103 | 0.115 | -0.122 | 0.328 | 0.369 |
| C(Smoking)[T.1] | 0.055 | 0.054 | -0.05 | 0.161 | 0.305 |
| C(Smoking)[T.2] | -0.022 | 0.058 | -0.136 | 0.091 | 0.697 |
| C(Surgical_Approach)[T.1] | -0.044 | 0.071 | -0.183 | 0.096 | 0.538 |
| C(Resection_Type)[T.2] | 0.062 | 0.052 | -0.039 | 0.163 | 0.226 |
| Group | -0.169 | 0.046 | -0.259 | -0.079 | <0.001 |
| Age | -0.004 | 0.004 | -0.012 | 0.004 | 0.293 |
| Sex | 0.036 | 0.043 | -0.048 | 0.12 | 0.406 |
| BMI | -0.002 | 0.007 | -0.016 | 0.011 | 0.721 |
| Preop_Pain | -0.008 | 0.023 | -0.053 | 0.037 | 0.723 |
| Operation_Time | 0.001 | 0.001 | -0.001 | 0.002 | 0.529 |
| Regional_Analgesia | -0.053 | 0.044 | -0.14 | 0.033 | 0.225 |
| NSAIDs | -0.026 | 0.044 | -0.112 | 0.059 | 0.545 |
