# Supplementary material for: Effect of ERAS-based refined nursing on postoperative pain management in lung cancer surgery patients
Source: Front Surg. 2026 May 28;13:1808117. doi: 10.3389/fsurg.2026.1808117 (PMC13254267; doi:10.3389/fsurg.2026.1808117)
Supplement: Supplementary file 5 [file Table5.docx]

**Supplementary Table S5.** Total-effect and exploratory direct-effect multivariable logistic regression models for rescue analgesia.

| **Term** | **OR** | **SE** | **95% CI (low)** | **95% CI (high)** | **p value** |
| --- | --- | --- | --- | --- | --- |
| Intercept | 0.45 | 3.186292 | 0 | 230.2 | 0.8 |
| C(ASA)[T.2] | 0.96 | 0.667737 | 0.26 | 3.57 | 0.956 |
| C(ASA)[T.3] | 0.88 | 0.991511 | 0.13 | 6.17 | 0.901 |
| C(Smoking)[T.1] | 0.82 | 0.475561 | 0.32 | 2.07 | 0.667 |
| C(Smoking)[T.2] | 0.57 | 0.52437 | 0.2 | 1.6 | 0.286 |
| C(Surgical_Approach)[T.1] | 1.29 | 0.701827 | 0.33 | 5.12 | 0.713 |
| C(Resection_Type)[T.2] | 1.06 | 0.530873 | 0.37 | 2.99 | 0.916 |
| Group | 0.37 | 0.447785 | 0.16 | 0.9 | 0.028 |
| Age | 1.02 | 0.037693 | 0.95 | 1.1 | 0.551 |
| Sex | 0.27 | 0.423081 | 0.12 | 0.62 | 0.002 |
| BMI | 1.06 | 0.072321 | 0.92 | 1.22 | 0.457 |
| Preop_Pain | 0.96 | 0.202759 | 0.64 | 1.42 | 0.823 |
| Operation_Time | 0.99 | 0.00774 | 0.98 | 1.01 | 0.301 |
| Regional_Analgesia | 1.12 | 0.40197 | 0.51 | 2.47 | 0.775 |
| NSAIDs | 0.33 | 0.430221 | 0.14 | 0.76 | 0.01 |
