# Supplementary material for: Effect of ERAS-based refined nursing on postoperative pain management in lung cancer surgery patients
Source: Front Surg. 2026 May 28;13:1808117. doi: 10.3389/fsurg.2026.1808117 (PMC13254267; doi:10.3389/fsurg.2026.1808117)
Supplement: Supplementary file 6 [file Table6.docx]

**Supplementary Table S6.** Total-effect and exploratory direct-effect multivariable logistic regression models for moderate-to-severe pain on POD2–POD3.

| **Term** | **OR** | **SE** | **95% CI (low)** | **95% CI (high)** | **p value** |
| --- | --- | --- | --- | --- | --- |
| Intercept | 0.25 | 6.896167 | 0 | 183293.2 | 0.839 |
| C(ASA)[T.2] | 0.41 | 1.494061 | 0.02 | 7.66 | 0.551 |
| C(ASA)[T.3] | 3.58 | 1.895537 | 0.09 | 146.96 | 0.501 |
| C(Smoking)[T.1] | 0.49 | 1.052126 | 0.06 | 3.89 | 0.504 |
| C(Smoking)[T.2] | 1.19 | 0.958069 | 0.18 | 7.79 | 0.856 |
| C(Surgical_Approach)[T.1] | 1.41 | 1.240154 | 0.12 | 16.03 | 0.781 |
| C(Resection_Type)[T.2] | 4.13 | 0.938782 | 0.66 | 25.98 | 0.131 |
| Group | 0.33 | 1.009074 | 0.05 | 2.41 | 0.276 |
| Age | 0.98 | 0.081852 | 0.83 | 1.15 | 0.8 |
| Sex | 0.91 | 0.902505 | 0.16 | 5.34 | 0.918 |
| BMI | 1.03 | 0.160594 | 0.75 | 1.41 | 0.846 |
| Preop_Pain | 1.02 | 0.435891 | 0.44 | 2.41 | 0.957 |
| Operation_Time | 1.01 | 0.016799 | 0.97 | 1.04 | 0.661 |
| Regional_Analgesia | 0.13 | 0.946614 | 0.02 | 0.85 | 0.033 |
| NSAIDs | 0.12 | 0.919638 | 0.02 | 0.72 | 0.02 |
