# Supplementary material for: Effect of ERAS-based refined nursing on postoperative pain management in lung cancer surgery patients
Source: Front Surg. 2026 May 28;13:1808117. doi: 10.3389/fsurg.2026.1808117 (PMC13254267; doi:10.3389/fsurg.2026.1808117)
Supplement: Supplementary file 7 [file Table7.docx]

**Supplementary Table S7.** Model fit indices for all multivariable models.

| **Model** | **N** | **R2 / PseudoR2** | **AIC** | **BIC** |
| --- | --- | --- | --- | --- |
| AUPC (OLS HC3) | 164 | 0.255 | 759.3 | NA |
| log(MME+1) (OLS HC3) | 164 | 0.231 | 350.5 | NA |
| log(LOS) (OLS HC3) | 164 | 0.162 | 32.1 | NA |
| Rescue analgesia (logit) | 164 | 0.143 | 191.8 | NA |
| Moderate-to-severe pain on POD2–POD3 (logit) | 164 | 0.28 | 84.2 | NA |
| Pain trajectory LMM (random intercept only) | 656 | NA | 1650.77 | 1736.01 |
| Pain trajectory LMM (random intercept and random slope) | 656 | NA | 1645.55 | 1739.76 |
