# Supplementary material for: Effect of ERAS-based refined nursing on postoperative pain management in lung cancer surgery patients
Source: Front Surg. 2026 May 28;13:1808117. doi: 10.3389/fsurg.2026.1808117 (PMC13254267; doi:10.3389/fsurg.2026.1808117)
Supplement: Supplementary file 8 [file Table8.docx]

**Supplementary Table S8.** Core components, responsible personnel, delivery frequency, and implementation fidelity of the ERAS-based refined nursing pathway.

| **Component** | **Planned timing** | **Responsible personnel** | **Documentation source** | **Delivered / eligible patients, n/N** | **Fidelity rate, %** |
| --- | --- | --- | --- | --- | --- |
| Preoperative education on anticipated pain trajectory, coping strategies, and mobilization goals | Preoperative period | Ward nurses | Nursing education record / admission nursing notes | 70/70 | 100.0 |
| Protocolized postoperative pain assessment with complete POD0–POD3 recording | POD0–POD3 | Ward nurses | Nursing pain record | 70/70 | 100.0 |
| Timely communication with surgical and anesthesia teams in case of insufficient analgesia | POD0–POD3, when indicated | Ward nurses, surgical team, anesthesia team | Nursing progress notes / consultation record | 70/70 | 100.0 |
| Scheduled administration of nonopioid analgesics according to institutional standards unless contraindicated | POD0–POD3 | Ward nurses / attending physicians | Medication administration record | 54/70 | 77.1 |
| Coordination of regional analgesia when applied | Perioperative to early postoperative period | Anesthesia team / ward nurses | Anesthesia / perioperative record | 43/70 | 61.4 |
| Coordination of patient-controlled analgesia when applied | Early postoperative period | Anesthesia team / ward nurses | Postoperative analgesia order / nursing record | 45/70 | 64.3 |
| Early mobilization instruction and related documentation | Beginning on POD1 | Ward nurses | Nursing progress notes / ambulation record | 70/70 | 100.0 |
| Progressive activity goal reinforcement | POD1 onward | Ward nurses | Nursing progress notes | 62/70 | 88.6 |
| Respiratory rehabilitation support, including incentive spirometry instruction | POD1 onward | Ward nurses | Nursing progress notes / respiratory training record | 54/70 | 77.1 |
| Effective coughing guidance and airway clearance support | POD1 onward | Ward nurses | Nursing progress notes | 23/70 | 32.9 |
| Proactive symptom co-management for nausea and vomiting | Postoperative period, when indicated | Ward nurses | Nursing notes / symptom record | 12/70 | 17.1 |
| Proactive symptom co-management for sleep disturbance | Postoperative period, when indicated | Ward nurses | Nursing notes / symptom record | 16/70 | 22.9 |
| Proactive symptom co-management for constipation-related discomfort | Postoperative period, when indicated | Ward nurses | Nursing notes / symptom record | 37/70 | 52.9 |

Fidelity rate was calculated as the number of ERAS-group patients with documented delivery of the corresponding pathway component divided by the number of ERAS-group patients eligible for analysis. The counts reported in this table reflect documented nursing delivery or pathway-related management records, which are not necessarily identical to the number of patients with corresponding postoperative events or symptoms reported in the main outcome tables.
