# Supplementary material for: Effect of ERAS-based refined nursing on postoperative pain management in lung cancer surgery patients
Source: Front Surg. 2026 May 28;13:1808117. doi: 10.3389/fsurg.2026.1808117 (PMC13254267; doi:10.3389/fsurg.2026.1808117)
Supplement: Supplementary file 9 [file Table9.docx]

**Supplementary Table S9.** Covariate balance before and after IPTW.

| **Variable** | **Unweighted SMD** | **Weighted SMD** |
| --- | --- | --- |
| Age | 0.134 | 0.048 |
| BMI | 0.018 | 0.045 |
| Charlson comorbidity index | 0.105 | 0.03 |
| Preoperative pain score | 0.1 | 0.022 |
| Sex | 0.157 | 0.026 |
| Hypertension | 0.213 | 0 |
| Diabetes | 0.072 | 0.069 |
| COPD | 0.274 | 0.001 |
| Preoperative analgesic use | 0.176 | 0.04 |
| Surgical approach | 0.248 | 0.077 |
| Resection type | 0.111 | 0.022 |
| Smoking = Never | 0.078 | 0.027 |
| Smoking = Former | 0.226 | 0.054 |
| Smoking = Current | 0.346 | 0.029 |
| ASA class I | 0.116 | 0.014 |
| ASA class II | 0.142 | 0.027 |
| ASA class III | 0.051 | 0.025 |
