# Supplementary material for: Effect of ERAS-based refined nursing on postoperative pain management in lung cancer surgery patients
Source: Front Surg. 2026 May 28;13:1808117. doi: 10.3389/fsurg.2026.1808117 (PMC13254267; doi:10.3389/fsurg.2026.1808117)
Supplement: Supplementary file 10 [file Table10.docx]

**Supplementary Table S10.** IPTW sensitivity analyses for the primary and key secondary outcomes.

| **Outcome** | **Model** | **Key effect** | **Effect size** | **95% CI** | **p value** |
| --- | --- | --- | --- | --- | --- |
| Postoperative pain trajectory (POD0–POD3) | Weighted GEE | Group × Time | -0.323 | -0.429 to -0.217 | <0.001 |
| AUPC (POD0–POD3) | Weighted linear regression | Group | -0.955 | -1.727 to -0.183 | 0.015 |
| Total opioid consumption (log[MME+1]) | Weighted linear regression | Group | -0.419 | -0.666 to -0.172 | <0.001 |
| Postoperative length of stay (log[LOS]) | Weighted linear regression | Group | -0.199 | -0.290 to -0.108 | <0.001 |
| Rescue analgesia (Yes vs No) | Weighted logistic regression | Group | 0.31 (OR) | 0.14 to 0.70 | 0.005 |
| Moderate-to-severe pain on POD2–POD3 (≥4) | Weighted logistic regression | Group | 0.28 (OR) | 0.05 to 1.52 | 0.140 |
